# Determination of GLUT1 Oligomerization Parameters using Bioluminescent Förster Resonance Energy Transfer

**DOI:** 10.1038/srep29130

**Published:** 2016-06-30

**Authors:** Brendan Looyenga, Calvin VanOpstall, Zion Lee, Jed Bell, Evans Lodge, Katherine Wrobel, Eric Arnoys, Larry Louters

**Affiliations:** 1 Calvin College, Department of Chemistry & Biochemistry, 3201 Burton St SE, Grand Rapids, MI, 49546, USA.

## Abstract

The facilitated glucose transporter GLUT1 (SLC2A1) is an important mediator of glucose homeostasis in humans. Though it is found in most cell types to some extent, the level of GLUT1 expression across different cell types can vary dramatically. Prior studies in erythrocytes—which express particularly high levels of GLUT1—have suggested that GLUT1 is able to form tetrameric complexes with enhanced transport activity. Whether dynamic aggregation of GLUT1 also occurs in cell types with more modest expression of GLUT1, however, is unclear. To address this question, we developed a genetically encoded bioluminescent Förster resonance energy transfer (BRET) assay using the luminescent donor Nanoluciferase and fluorescent acceptor mCherry. By tethering these proteins to the N-terminus of GLUT1 and performing saturation BRET analysis, we were able to demonstrate the formation of multimeric complexes in live cells. Parallel use of flow cytometry and immunoblotting further enabled us to estimate the density of GLUT1 proteins required for spontaneous oligomerization. These data provide new insights into the physiological relevance of GLUT1 multimerization as well as a new variant of BRET assay that is useful for measuring the interactions among other cell membrane proteins in live cells.

The efficiency of glucose uptake into cells is a major determinant of bioenergetic homeostasis. Members of the facilitated glucose transporter/solute carrier 2A (GLUT/SLC2A) family of proteins passively transport glucose across the plasma membrane, primarily enabling glucose uptake from systemic circulation^1^. Within this family, GLUT1 and GLUT4 are the most well studied members due to their roles in basal and insulin-stimulated glucose uptake, respectively. While the mechanisms that regulate GLUT4 abundance at the cell membrane have been the subject of intense investigation, the mechanisms that govern GLUT1 abundance at the membrane have received less attention. Because of its relatively ubiquitous expression and its role in basal glucose uptake, it was long assumed that GLUT1 is primarily regulated by transcriptional expression, with little acute regulation at the level of cell signaling. More recent studies, however, suggest that the subcellular localization of GLUT1 is highly dynamic, albeit distinct from that of GLUT4^2^,^3^,^4^,^5^.

Several overlapping—and sometimes competing—mechanisms regulate the activity of GLUT1 in glucose transport. First among these is the differential expression of GLUT1 in distinct cell types, which can vary by orders of magnitude, for example in cancer versus normal cells^6^,^7^. The transcriptional programs that maintain the basal steady-state of GLUT1 expression in different cell types are poorly understood, though it is clear that GLUT1 is expressed to some degree in most cells of the body and plays an important role in basal glucose uptake. Various perturbations of metabolic homeostasis, including decreases in nutrient or oxygen availability, typically increase basal GLUT1 transcription, as do extracellular signals that promote cellular growth and proliferation^8^,^9^.

A second mechanism of GLUT1 activation involves the regulation of its subcellular distribution between internal vesicular compartments and the cell surface. Unlike the abundance of GLUT4 (SLC2A4) at the membrane, which is more binary in nature and acutely triggered by extracellular signals such as insulin, the abundance of GLUT1 on the cell surface appears to be continuously titrated in response to the bioenergetic status of the cell, particularly with regards to the critical variable of cytoplasmic [AMP]/[ATP] ratio^3^. Changes in this ratio differentially trigger the activity of AMP-activated kinase (AMPK), which in turn controls the activity of TXNIP, a protein that binds to the C-terminal domain of GLUT1 to trigger its endocytic internalization^10^. This same domain appears to be important for sorting, recycling and retention of GLUT1 in various cellular compartments, suggesting a complex interplay among several different proteins that differentially control the subcellular localization of GLUT1 under different physiological circumstances^2^,^5^.

A third mechanism that impinges on the activity of GLUT1 involves its ability to form higher-order multimeric complexes on the plasma membrane^11^,^12^. Work primarily performed in erythrocytes has demonstrated that GLUT1 monomers can associate into homodimeric and homotetrameric complexes that display enhanced transport activity relative to monomeric transporters^13^,^14^. While these studies provide compelling evidence for multimerization as mechanism of regulation, it is currently unclear whether it applies only to erythrocytes—which express unusually high levels of surface GLUT1—or can also be generally applied to other cell types with lower expression levels^15^.

An efficient and simple method to quantify the number of molecules required for physical interaction to occur at the plasma membrane involves the use of bioluminescent Förster resonance energy transfer (BRET) between a luciferase donor and a fluorescent acceptor^16^,^17^. In this proximity-dependent assay, the energy from a bioluminescent reaction catalyzed by luciferase is harnessed to stimulate the fluorescence of an acceptor fluorophore that is typically located within 10 nm of the luciferase donor. A number of donor/acceptor pairs for BRET assays have been previously described, the most common of which utilize *Renilla* luciferase (Rluc) and a green fluorescent protein (GFP) derivative such as the yellow fluorescent protein (YFP)^16^. The sensitivity of BRET assays depends heavily upon the degree of spectral overlap between donor emission and acceptor excitation. Although the sensitivity of the assay tends to increase along with greater spectral overlap, a greater degree of overlap also produces a higher signal-to-noise ratio that limits dynamic range of the assay^18^.

In this study we describe a new version of the traditional BRET assay using a small (19 kDa) monomeric luciferase derived from deep-sea shrimp (Nanoluciferase, Nluc) as a donor and the well-characterized red fluorescent protein mCherry as an acceptor^19^,^20^. Use of this new BRET pair overcomes some of the problems traditionally associated with BRET, as the donor and acceptor have little overlap in their emission spectra, yet can still produce a sensitive BRET signal due to the unusually high catalytic efficiency of Nluc^19^,^21^. Previous studies have demonstrated that Nluc is capable of exciting fluorophores that emit in the far-red spectrum, though this work utilized organic fluorophores rather than genetically encoded fluorescent proteins^21^,^22^. By tethering Nluc and mCherry proteins to either terminus of GLUT1, we were able to demonstrate the formation of multimeric complexes in live cells, while also confirming the previously described orientation of GLUT1 monomers within this complex. Single-molecule BRET with dual tagged GLUT1 also allowed us to estimate the distance between N- and C-termini in GLUT1, while saturation BRET analysis coupled with flow cytometry allowed us to estimate the density of GLUT1 proteins required to achieve oligomerization. This approach provides a useful framework for validating biochemically defined protein interactions in live cells and also allows for a more precise way of determining the physiological relevance of these interactions in comparison to endogenous levels of protein expression in biological membranes.

## Results

### Validation of the Genetically-Encoded BRET Pair: NanoLuciferase and mCherry

The quantitative principles governing BRET reactions are based upon equations developed by Theodor Förster for determining resonance energy transfer between two fluorophores (FRET)^16^,^23^. For the purposes of determining the degree of association between molecules in the two-dimensional plane of a biological membrane, the two key factors of interest are the efficiency of resonance energy transfer (*E*) between a given donor/acceptor pair, and the distance at which this efficiency is at 50%, defined as the Förster radius (*R*_*0*_)^24^. These two factors are related by the Förster equation (equation 1), which specifies that BRET efficiency is proportional to the sixth power of the ratio between the actual distance between donor and acceptor (*R*) and the Förster radius.


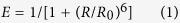


The Förster radius is a unique value for every donor/acceptor pair that can be calculated using four essential variables that are specific to a given BRET assay (equation 2). These variables include the orientation factor (κ) between the donor and acceptor, the quantum yield of the donor luciferase (Φ_0_), the refractive index of the assay medium (*n*), and the degree of overlap (*J*) between the donor emission spectrum and acceptor excitation spectrum.


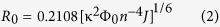


Assuming that both the donor and acceptor molecules in a given BRET assay are free from rotational constraints, a dipole orientation factor of 2/3 can be assigned for determination of the Förster radius^25^. The other three variables must be experimentally determined for specific donor/acceptor pairs.

**Figure 1:**
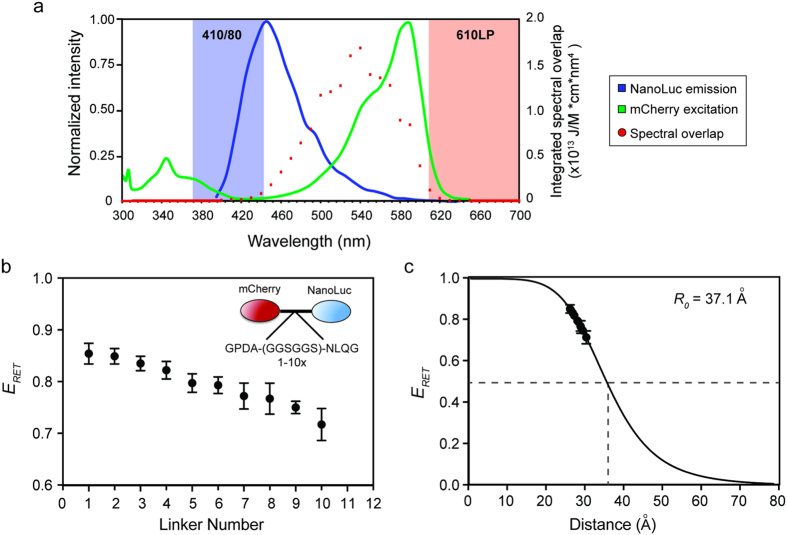
Determination of BRET parameters for the Nanoluciferase/mCherry combination. (**a**) The normalized emission spectrum of Nanoluciferase (NanoLuc) and excitation spectrum of mCherry are shown in relation to the filter sets used to detect emission from each molecule. Nluc emission was detected using a 410 nm peak filter with an 80 nm bandwidth (blue shading), while mCherry emission was detected with a 610 nm longpass filter (red shading). Both filter sets optimally detect light well outside of the overlap region in which bioluminescent resonance energy transfer occurs. (**b**) The resonance energy transfer efficiency (*E*_*RET*_, equation 3) was calculated from spectral emission data for a series of vectors in which mCherry and Nluc were tethered by different numbers of a hexapeptide repeat (inset) of known dimensions. Error bars represent standard deviation of averaged values across three separate experiments. **(c)** Fitting of RET efficiency values and physical distances between mCherry and Nluc to the Förster equation using a Förster radius of 37.1 angstroms (see Table 1) gives a line fit of *r*^*2*^ = 0.9995, demonstrating good correlation of experimental data to the theoretically predicted value.

Previous BRET pairs using firefly or *Renilla* luciferase suffer from a lack of dynamic range due to their use of donor/acceptor pairs with relatively poor spectral separation^16^. To improve this critical aspect of BRET, we reasoned that use of a brighter donor would allow us to use an acceptor with greater spectral separation without losing detectable BRET emission. To this end we chose Nanoluciferase (Nluc) as a donor due to the fact that is has a turnover rate more than 100 times higher than that of firefly luciferase (Fluc) or *Renilla* luciferase (Rluc)^19^. The reaction catalyzed by Nluc is spectrally similar to that of Rluc, emitting light in the blue spectrum with a peak at 460 nm (Fig. 1a). Furthermore, the quantum yield of this enzyme has previously been published, allowing us to calculate the theoretical Förster radius with a variety of red-shifted acceptor proteins with published extinction coefficients^20^,^26^. These data were used to generate a table of theoretical values for several monomeric BRET acceptors (Table 1)^25^.

Among the various monomeric fluorescent acceptor proteins we considered, we chose to work with mCherry as an acceptor due to its large spectral separation from Nluc (Table 1). Using mCherry provides an exceedingly low theoretical “background BRET ratio” (610 nm/410 nm for Nluc only), which is coupled with a reasonable fluorescent brightness compared to similarly red-shifted acceptors (Fig. 1a; Table 1). Though other fluorescent acceptors would potentially work as well or better in BRET assays with Nluc, use of mCherry as an acceptor emphasizes the particular utility of Nluc as a donor to significantly red-shifted proteins in BRET assays.

To empirically test the efficiency of the Nluc-mCherry BRET pair and validate its predicted Förster radius, we produced a series of mCherry-Nluc fusion proteins linked by a repeat peptide of known dimensions^24^. These proteins were transfected into 293FT-HEK cells in a 96-well format and assayed using a luminescence plate reader equipped with filters capable of separating the Nluc and mCherry spectra (Fig. 1a). Using this approach we determined the efficiency of resonance energy transfer (*E*_*RET*_) for each fusion construct according to equation 3, which relates the ratio of donor emission to acceptor emission (410/610 nm) in the presence of either the donor alone (*I*_*D*_) or both the donor and acceptor (*I*_*DA*_).


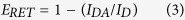


A plot of these data as a function of the number of hexapeptide linker repeats clearly demonstrates that increasing the number of repeats leads to a decrease in BRET efficiency, as would be predicted by the Förster equation (Fig. 1b).

The hexapeptide linker (GGSGGS) produces a tertiary fold of known diameter^24^, which further allowed us to fit the BRET efficiency data to the Förster equation along with calculated Förster radius of 37.1 angstroms (Table 1). Fitting of our measured efficiency data to this equation with actual distances (R) between Nluc and mCherry in our series of linker fusion proteins produced a highly significant correlation of 0.9995 (Fig. 1c). Importantly, the background signal of Nluc alone is extremely low (I_D_ = 0.0033 +/− 0.0003), which allows us to measure changes in BRET ratio without dampening due to high background noise (Supplemental Figure 1). These data provide strong empirical validity for the use of Nluc and mCherry as a viable donor/acceptor pair in BRET assays.

### Assignment of distance between the N- and C-termini of GLUT1 using BRET

**Figure 2:**
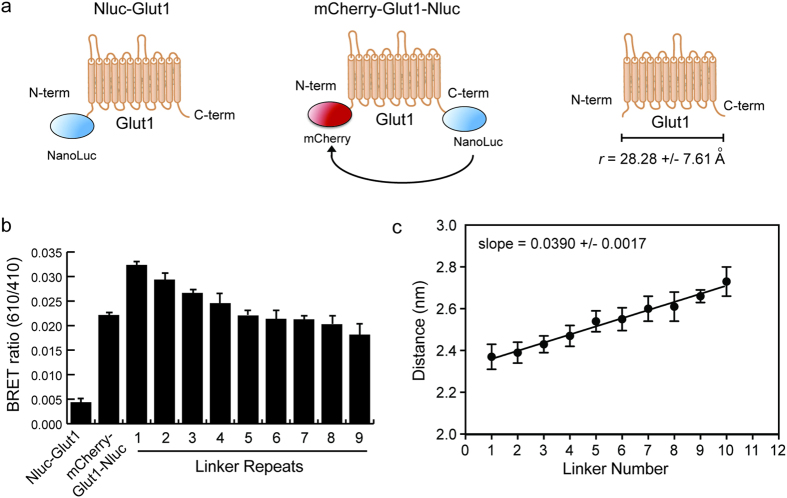
Determination of the distance between N- and C-termini of GLUT1 using BRET. **(a)** Cartoon diagrams of the vectors used for determination of BRET values for dual-labeled GLUT1. The background BRET ratio (610 nm/410 nm signal) was determined using GLUT1 fused to Nluc alone, while a dual-tagged vector with mCherry linked to the N-terminus and Nluc linked to the C-terminus was used to measure the average distance between these two sites. **(b)** Absolute BRET ratios are shown for each of the indicated fusion constructs, with error bars representing standard deviations of triplicate experiments. The BRET ratio for the dual-tagged GLUT1 lies within the range of values determined for the linker vector series. **(c)** The linker vector series was used to create a calibration curve relating distance to linker number, and therefore BRET ratio. Fitting of the data derived from the dual-tagged GLUT1 vector to this curve produced an estimated distance of 28.28 +/− 7.61 angstroms between the N- and C-termini of GLUT1.

The hexapeptide linker series we created produces data that fit well in the linear portion of the Förster equation (equation 1), allowing us to produce a standard curve by which the distance between Nluc and mCherry can be calculated using linkers of unknown dimensions (Fig. 2c). With this principle in mind, we fused mCherry to the N-terminus of the glucose transporter GLUT1 and Nluc to the C-terminus to create a fusion protein in which the donor and acceptor interact at a fixed distance in transfected cells (Fig. 2a). The BRET ratio (610/410 nm) created by this construct fit within the calibrated hexapeptide linker series we had previously tested, allowing us to determine the average distance between the N- and C-termini of GLUT1. Our data suggest an average distance of 28.28 +/− 7.61 angstroms typically separates these two domains when GLUT1 is inserted into the membrane of cells (Fig. 2b), which is a reasonable estimate based on the crystal structure and homology model of human GLUT1^27^,^28^.

### Estimation of molecular density required for oligomerization of GLUT1 on the plasma membrane

**Figure 3:**
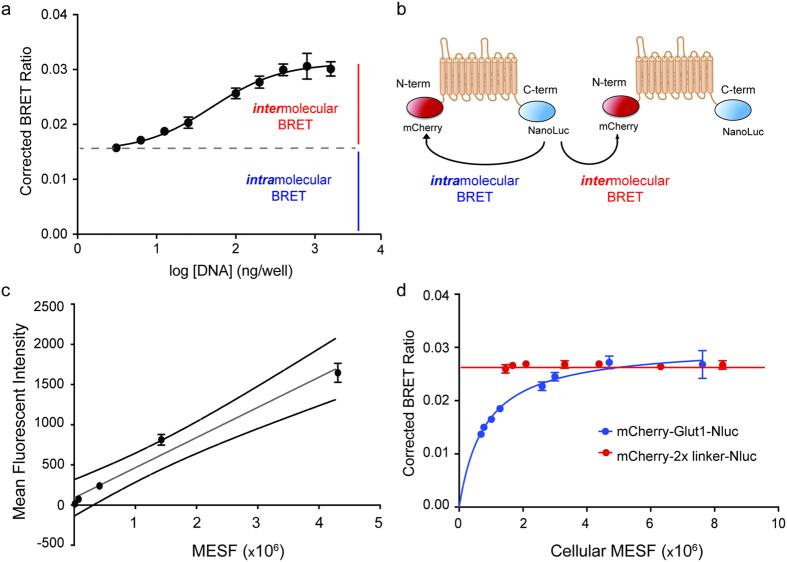
Saturation BRET analysis with dual-tagged GLUT1 indicates the formation of higher-order homomeric complexes. **(a)** Transfection of increasing amounts of plasmid vector encoding the GLUT1 dual-tagged vector produces a saturable increase in corrected BRET values, determined by subtracting the Nluc-GLUT only BRET ratio from the absolute BRET ratio for each experimental replicate. Error bars represent standard deviation of averaged values across three separate experiments (*R*^*2*^ = 0.996). **(b)** Cartoon diagram of distinct modes of BRET that are proposed to interpret the non-linear increase in BRET signal associated with increasing expression of mCherry-GLUT1-Nluc. Saturability is indicative of resonant energy transfer between aggregated fusion proteins (intermolecular BRET), and occurs in addition to the local transfer of energy between the N- and C-termini of monomers (intramolecular BRET). **(c)** Standard curve of fluorescent intensity versus MESF (molecular equivalents of soluble fluorophore) for mCherry beads, which was used to estimate the number of mCherry-Glut1-Nluc fusion proteins expressed in 293FT cells by transient transfection. External lines (black) represent 95% confidence intervals for the line-fitted data (gray, *R*^*2*^ = 0.9611). Error bars represent standard deviations of four separate cytometry runs using identical laser settings on the cytometer. **(d)** Comparison of BRET values and molecular equivalent of soluble fluorophore (MESF) values derived from cells transfected with the dual-tagged GLUT1 (blue, *R*^*2*^ = 0.949) versus those transfected with a mCherry-Nluc fusion vector (red, mCherry-2x linker-Nluc). Error bars represent standard deviation of averaged values across three separate experiments.

We noticed that the BRET signal produced by our mCherry-GLUT1-Nluc fusion protein increased as a function of increasing plasmid transfection (Fig. 3a). This finding was unexpected since the BRET ratio for mCherry-Nluc fusion proteins typically remains constant regardless of the amount of vector transfected into cells due to the 1:1 stoichiometry of donor:acceptor and fixed distance between them. In contrast, the GLUT1 dual fusion protein demonstrates saturation kinetics that would be expected of a fixed amount of donor being titrated with increasing concentrations of acceptor (Fig. 3a).

To explain this unexpected result, we hypothesize that two modes of resonance energy transfer are occurring in cells transfected with mCherry-GLUT1-Nluc. The corrected BRET ratio produced at low levels of expression reflects “intramolecular BRET” between the N- and C-termini of GLUT1 molecules, which remains constant when mCherry-GLUT1-Nluc exists in a monomeric state. In contrast, the increased BRET signal seen with increasing expression levels of mCherry-GLUT1-Nluc can be accounted for by “intermolecular BRET” between molecules of a multimer in which light emitted from the Nluc donor on one mCherry-GLUT1-Nluc protein activates the fluorescence of mCherry on a juxtaposed fusion protein (Fig. 3b). These data would explain the saturation kinetics of our data and can be further confirmed using co-transfection of individually labeled GLUT1 proteins.

Given the saturation kinetics we observed for intermolecular BRET with the dual-labeled GLUT1 fusion protein, we hypothesized that it might be possible to estimate the number of GLUT1 molecules required to induce multimerization on the surface of 293FT cells. In this system, the number of mCherry molecules is equivalent to that of the GLUT1 proteins to which they are fused, indicating that accurate quantification of mCherry abundance relative to the BRET ratio would provide an estimate of the number of fusion proteins necessary to induce intermolecular BRET, which serves as a proxy for oligomerization. To quantify the average number of mCherry-GLUT1-Nluc proteins present in cells after transfection, we produced a standard curve using mCherry-coated beads whose fluorescence could be measured by flow cytometry (Fig. 3c). These beads are calibrated to specific MESF (molecular equivalent of soluble fluorophore) values, which provide a correlation between fluorescent intensity and molecular abundance.

Using the settings derived from mCherry beads, we then measured the fluorescent intensity of cells transfected with different amounts of the mCherry-GLUT1-Nluc. After converting the fluorescent intensities of each cell population to an MESF value, we plotted each MESF against corrected BRET ratios (BRET signal minus Nluc-only signal) and fit them to a hyperbolic saturation curve (Fig. 3d). These data produced an EC_50_ value of 8.40 × 10^5^+/−1.58 × 10^5^ molecules/cell, suggesting that approximately this number of GLUT1 proteins must be expressed in a 293FT cells in order for 50% of the molecules to be involved in multimeric complexes. Importantly, parallel experiments with a mCherry-2xlinker-Nluc fusion protein connected by two hexapeptide repeats failed to display saturation BRET kinetics, and instead produced a linear curve fit in which the BRET ratio remained the same regardless of the number of fusion protein expressed (Fig. 3d). These data support the hypothesis that mCherry-GLUT1-Nluc forms higher-order multimers in the membrane, while other soluble mCherry-Nluc fusion proteins do not.

### Validation of intermolecular BRET with individually tagged GLUT1 monomers

**Figure 4:**
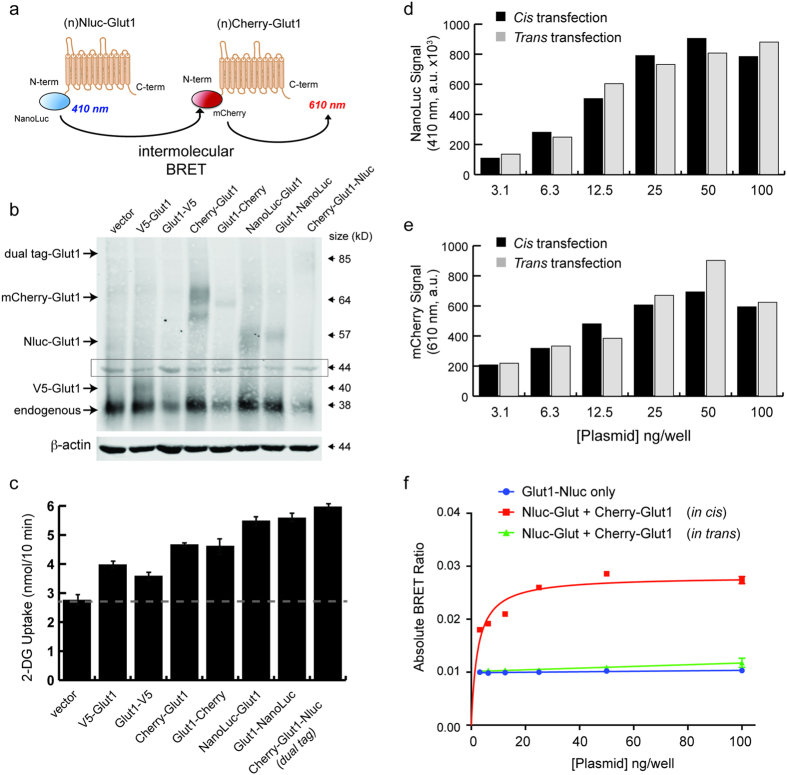
Validation of intermolecular BRET with separate GLUT1 fusion proteins. **(a)** Cartoon diagrams of the vectors used for determination of BRET values with individually labeled GLUT1 monomers fused to either Nluc or mCherry at the N-terminus. **(b)** Transient expression of the various GLUT1 fusion proteins in HEK-293FT cell lysates. The size of each fusion protein is consistent with expected molecular weights relative to the indicated mass ladder. Area in the box at 44 kilodaltons represents signal from β-actin (680 nm channel) that bled over into the Glut1 scan (800 nm channel). **(c)** Uptake of 2-deoxyglucose by HEK-293FT cells that were transfected with the indicated GLUT1 fusion proteins. Uptake assays were performed at a density of 5.0 × 10^4^ cells/well at 72 after transfection. **(d)** Raw 410/80 nm signals are shown for cells that were transiently transfected with increasing amounts of Nluc-GLUT1 and mCherry-GLUT1. Transfection in *cis* represents cells that received both vectors simultaneously, while transfection in *trans* represents a 50:50 mix of cells that received only one of the two vectors transfected separately. Values represent the average of duplicate wells. **(e)** Raw mCherry fluorescence values (Ex: 585 nm, Em: 610 nm, monochrometer) are shown for the same cells as in D. Values represent the average of duplicate wells. **(f)** Absolute BRET ratios are shown for the same cells as in D. Actual resonance energy transfer is only occurring in cells that display BRET ratios above the background for Nluc only.

To further confirm that intermolecular BRET could be used to investigate the multimerization kinetics of GLUT1, we individually fused either Nluc or mCherry to the N-terminus of GLUT1 (Fig. 4a). When expressed in 293FT-HEK cells, these constructs produced single proteins at the predicted molecular weight, which could be detected along with the endogenous GLUT1 protein expressed by these cells (Fig. 4b). Importantly, all of the various fusion proteins increased 2-deoxyglucose transport into transfected cells, indicating that they are functionally active and properly transported to the plasma membrane (Fig. 4c).

To demonstrate that the BRET signals detected by co-transfection of individually tagged proteins result from physical interactions within a single membrane, we performed a control experiment in which the same amount of plasmid DNA for Nluc-GLUT1 and mCherry-GLUT1 were transfected into either the same 293FT cells (*cis* transfection) or into two separate cell populations that were subsequently mixed at a 1:1 ratio (*trans* transfection). Using filtered luminescence on our plate reader, we then measured the donor (410/80 nm filter) and acceptor (610 nm long-pass filter) emission signals generated by addition of the Nluc substrate furimazine. In addition we used the standard monochromater function on the same plate reader to measure mCherry fluorescence after excitation with 587 nm light. As expected, we observed a dose-dependent increase in total Nluc emission and mCherry fluorescence as a function of the amount of DNA transfected into each well (Fig. 4d,e). Using filtered luminescence measurement of the BRET signal produced by these experiments, however, we only detected a dose-dependent increase in BRET for the cells transfected in *cis*, whereas the cells transfected in *trans* displayed the same BRET ratio as observed for cells transfected with Nluc-GLUT1 only (Fig. 4f).

To further validate these findings, we also measured the complete emission spectrum of Nluc-Glut1 in the presence or absence of a doxycycline-inducible mCherry-Glut1 fusion protein using the monochromator function on our plate reader (Supplemental Figure 1a). When the mCherry-Glut1 is present, an upward shift in signal is seen in the region corresponding to the emission spectrum of mCherry (Fig. 1a, Supplemental Figure 1b). Disruption of cell membrane complexes using detergents has no effect on Nluc activity, but results in a clear loss of resonant energy transfer to mCherry, demonstrating the requirement of membrane co-localization for BRET to occur (Supplemental Figure 1c–e). These data indicate that intermolecular BRET signals from separately tagged GLUT1 molecules represent true physical interactions between GLUT1 monomers rather than non-specific activation of the acceptor by light scattering within the well plate assay.

### Determination of monomer orientation in GLUT1 multimers

Prior studies of GLUT1 in human erythrocytes have suggested that GLUT1 monomers interact in a specific orientation to form dimers, which then further organize into tetramers^11^,^14^. While the specific structural features of these higher order complexes have not been solved, studies with GLUT1/GLUT4 chimeras indicate that GLUT1 dimers form via interactions between the N-termini of juxtaposed monomers, likely at the interface between transmembrane helices 5 and 2^11^. In contrast, work with GLUT1/GLUT3 chimeras suggests that the formation of tetramers seems to involve C-terminal interactions mediated at least in part by transmembrane helix 9^12^. Because the maximum BRET ratio (BRET_max_) that can be achieved is a function of distance between the donor and acceptor, we reasoned that saturation BRET assays using different combinations of Nluc and mCherry tagged GLUT1 could be used to test this model of GLUT1 oligomerization^16^.

**Figure 5:**
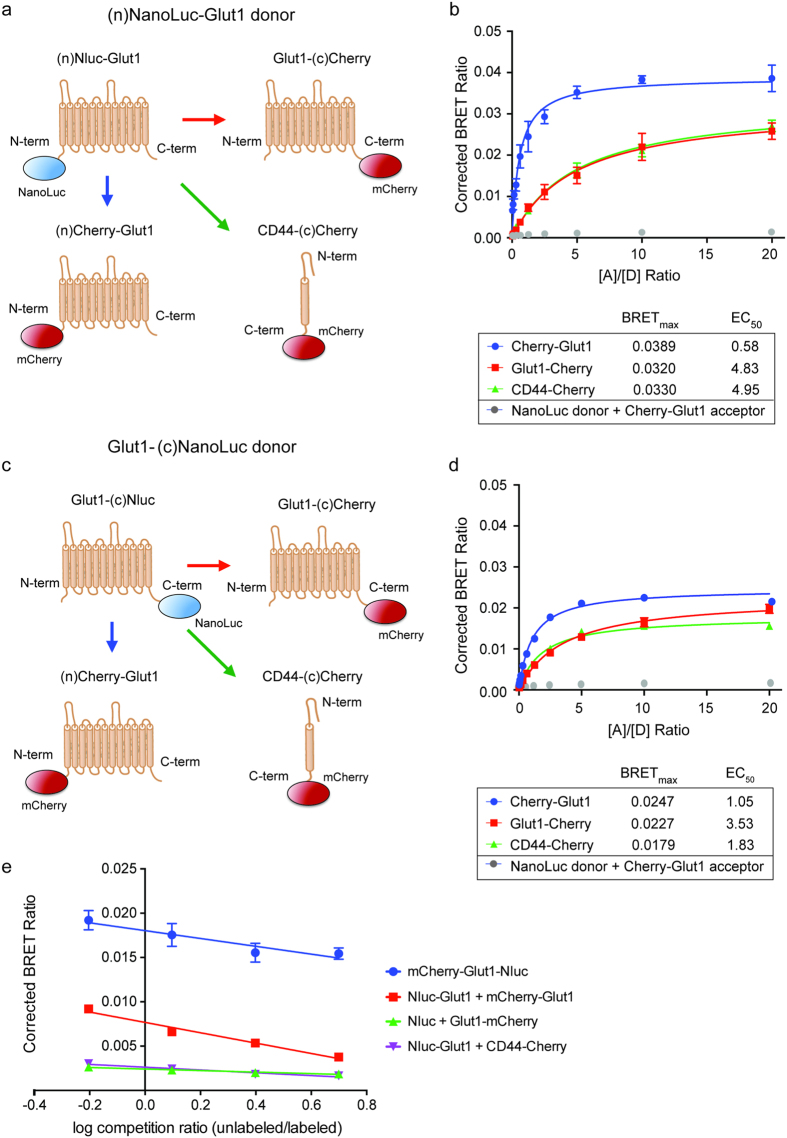
Intermolecular BRET between GLUT1 monomers suggests N-to-N terminal orientation occurs first in higher order complexes. **(a)** Cartoon diagrams of the vectors used in panel B for determination of BRET values with individually labeled GLUT1 monomers. Nluc was fused to the N-terminus of GLUT1 as donor, whereas the mCherry acceptor was fused to either terminus of GLUT1 or to the intracellular C-terminus of CD44 as a non-specific control. **(b)** Corrected BRET values are shown as a function of acceptor: donor ratio [A]/[D] in terms of plasmid concentration. The data for each acceptor/donor combination were fit to standard binding curves, from which the maximum BRET ratio (*BRET*_*max*_) and effective concentration at 50% *BRET*_*max*_ (EC_50_) could be determined in terms of plasmid ratios. *BRET*_*max*_ values are proportional to the distance between donor and acceptor, whereas EC_50_ values represent the relative affinity between donor and acceptor. As a negative control, soluble Nluc was co-transfected with mCherry-GLUT1 (gray data points) to demonstrate the necessity of membrane localization for BRET to occur. Error bars represent standard deviation of averaged triplicate values for a representative experiment. **(c)** Cartoon diagrams of the vectors used in panel D, using Nluc fused to the C-terminus of GLUT1 as the BRET donor. **(d)** Corrected BRET values are shown as a function of acceptor:donor ratio [A]/[D] in terms of plasmid concentration, as in panel B. **(e)** Corrected BRET values for an unlabeled competition assay are shown as a function of the ratio between unlabeled GLUT1, and the combination of Nluc-GLUT1 and mCherry-GLUT1. The intermolecular BRET signal produced by transfecting the donor and acceptor as separate fusion proteins (red line) could be competed down to background levels of signal (green and purple lines), whereas the BRET signal produced using the mCherry-GLUT1-Nluc dual fusion protein (blue line) could only be competed down to the level generated by intramolecular BRET between the N- and C-termini of GLUT1.

To perform saturation BRET assays, we transiently transfected 293FT cells with a fixed amount of Nluc-GLUT1 and increasing amounts of mCherry-GLUT1, while varying the location of the epitope tags on either terminus of the recombinant GLUT1 fusion proteins (Fig. 5a,c). As controls, we also performed the saturation BRET transfections with soluble mCherry only and a generic membrane protein (CD44-mCherry), which were predicted to produce no change in BRET ratio or an increase in signal due to non-specific membrane saturation, respectively. The data for each acceptor/donor combination were fit to standard binding curves, from which the maximum BRET ratio (*BRET*_*max*_) and effective concentration at half *BRET*_*max*_(EC_50_) could be determined in terms of plasmid ratios. In this context, *BRET*_*max*_ values are proportional to the distance between donor and acceptor, whereas EC_50_ values represent the relative affinity between donor and acceptor. These experiments clearly demonstrate that the closest association and highest relative affinity between Nluc and mCherry are achieved by transfecting cells with GLUT1 monomers bearing both fusion tags on N-termini (Fig. 5b). Furthermore, adding the mCherry acceptor to the C-terminus of GLUT1 had little more effect than when GLUT1-Nluc fusion proteins were transfected with the non-specific acceptor protein CD44-mCherry, suggesting that GLUT1 oligomers primarily form by orienting their N-termini together in close apposition (Fig. 5b,d).

As expected, the BRET interactions between these individually tagged GLUT1 proteins could be completely competed away by co-transfecting increasing amounts of unlabeled GLUT1 along with the donor and acceptor fusion proteins. In contrast, co-transfection of unlabeled GLUT1 with the dual-tagged mCherry-GLUT1-Nluc fusion protein only decreased its BRET signal to that seen with monomeric concentrations of the protein (Fig. 5e). Together these data support the idea that individual GLUT1 monomers can interact in stable, homomeric complexes if expressed at sufficient levels, and that these complexes primarily form via N-terminal interactions with each other.

### Stable saturation BRET analysis of GLUT1 complexes in renal cells

**Figure 6:**
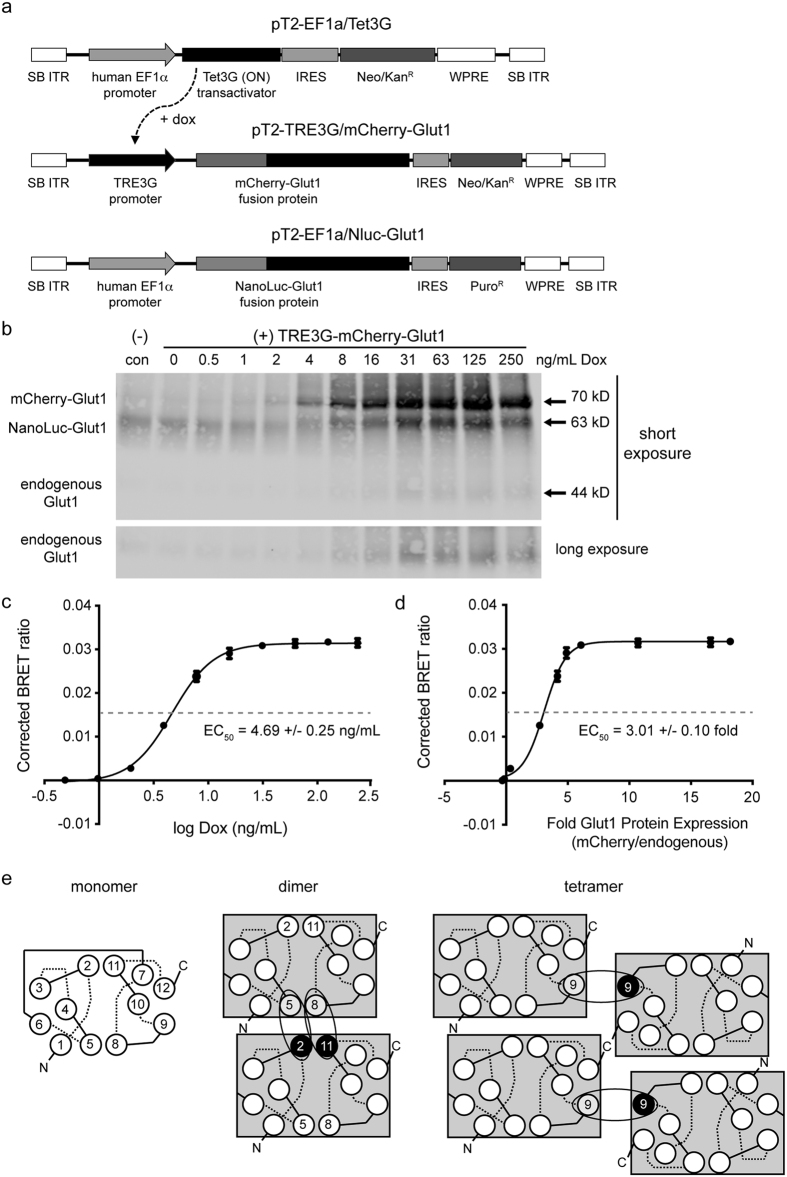
Determination of the relative GLUT1 density required for the formation of higher order membrane complexes. **(a)** Maps of the Sleeping Beauty transposable element vectors used for stable integration of Nluc-GLUT1 and a tetracycline-inducible mCherry-GLUT1 into transfected cells. **(b)** Stable 293FT/Nluc-GLUT1 (control) or inducible 293FT/dox^GB1^ cells were induced for 48 hours with the indicated concentration of doxycycline (Dox). Cell lysates were harvested, deglycosylated and separated by SDS-PAGE prior to immunoblotting for GLUT1. All three species of GLUT1 can be detected on blots based upon their differential sizes. Two separate exposure times are shown to demonstrate the differential abundance of the transgenic GLUT1 fusion proteins, and the endogenous GLUT1. **(c)** Corrected BRET ratios were plotted relative to the concentration of doxycycline used to induce mCherry-GLUT1 expression. Data were log fit to a sigmoidal curve (*R*^*2*^ = 0.999), from which a doxycycline EC_50_ value of 4.69 +/− 0.25 ng/mL was determined. **(d)** The fluorescent intensity of each band on the immunoblot in (B) was normalized to actin to derive a corrected pixel value. These values were then expressed as fold increases relative to endogenous GLUT1 for each sample. Corrected BRET ratios were plotted against relative protein expression values for GLUT1, and fit to a sigmoidal curve as indicated (*R*^*2*^ = 0.996) to obtain an EC_50_ value of 3.01 +/− 0.10 fold expression relative to endogenous GLUT1 in 293FT cells. Error bars represent standard deviations of triplicate experimental replicates. **(e)** Model of higher order GLUT1 complexes represented with interactions between monomers. Each monomer is shown with its twelve alpha helical transmembrane domains, with extracellular (dashed) and intracellular (solid) loop regions. Our BRET data are consistent with previous studies indicating that GLUT1 transporters initially associate as N-to-N terminal dimers with contact interfaces between helicies 5 and 8 of one monomer and helicies 2 and 11 of the other. Two sets of dimers then associate into a tetramer via interfaces involving helix 9 from each monomer. This orientation limits further associations and specifies tetramers as the preferred species under saturation conditions.

Having confirmed with our BRET system that GLUT1 monomers can form higher order complexes that preferentially associate via N-terminal interactions, we further determined the relative amount of GLUT1 required to achieve this interaction. To perform the saturation BRET assay between Nluc-GLUT1 and mCherry-GLUT1, we utilized the Tet3G inducible expression system engineered into a Sleeping Beauty (SB) transposable element vector (pT2) to allow for stable expression in 293FT-HEK cells^29^,^30^. In this system, Nluc-GLUT1 and the Tet3G transactivator protein are both constitutively expressed at moderate levels using the human elongation factor 1-alpha (EF1α) promoter (Fig. 6a), which is less prone to epigenetic silencing than the CMV promoter found in the pcDNA3.1 vector we used for transient transfection. Expression of mCherry-GLUT1 was in turn controlled by the Tet3G responsive element (TRE) promoter, which is bound and transcriptionally activated by Tet3G in a dose-responsive fashion by addition of the tetracycline analog doxycycline (Fig. 6a, Supplemental Figure 1a). These three vectors were co-transfected into 293FT cells along with the SB transposase, which allows for stable insertion of the expression cassette flanked by inverted terminal repeats (ITR) that this enzyme recognizes (Fig. 6a).

After selection of stable polyclonal cell lines (293FT/dox^GB1^) with puromycin, we induced expression of mCherry-GLUT1 with a 2-fold serial dilution of doxycycline for 48 hours to determine the amount of drug required to saturate cells with Glut1. Interestingly, we found that increased expression of the mCherry-GLUT1 fusion protein by doxycycline also resulted in accumulation of both Nluc-GLUT1 and endogenous GLUT1 (Fig. 6b). This finding suggests that the cellular machinery responsible for turnover of endogenous GLUT1 becomes progressively saturated with mCherry-GLUT1 and is unable to sustain removal of any of the three isoforms. As such, we were unable to use flow cytometry as a proxy for total Glut1 abundance, since the quantity of mCherry-Glut1 in cells is not directly proportional to the total abundance of Glut1 in cells.

As an alternative approach, we analyzed doxycycline-induced cells in parallel with immunoblot and BRET assay to determine the relative amount of mCherry-GLUT1 expression that would be required to form multimers capable of resonant energy transfer. The corrected BRET values were compared to concentrations of doxycycline (Fig. 6c) and to the relative amount of mCherry-GLUT1 protein (Fig. 6d), which was determined by comparing the pixel intensity of the mCherry-Glut1 band on immunblots to the pixel intensity of endogenous Glut1. Both data sets were then fit to hyperbolic saturation curves from which EC_50_ values could be calculated. Our data indicate that ~50% of mCherry-GLUT1 proteins are involved in higher order, multimeric complexes at 4.69 +/− 0.25 ng/mL of doxycycline, which corresponds to an expression level of about 3-fold over endogenous GLUT1 expressed in 293FT cells.

## Discussion

Development of simple, cost-effective assays for quantifying protein-protein interactions is critical for validating the wealth of data emerging from shotgun proteomic screens of affinity purified protein complexes. Bioluminescent Förster resonance energy transfer (BRET) has become a method of choice for studying protein-protein interactions between membrane proteins due to its ability to be measured in live cells using a simple plate reader format. Previous iterations of the methodology using firefly or *Renilla* luciferase BRET donors have demonstrated the broad utility of BRET for molecular and cellular biology studies, despite the relatively limited dynamic range of these assays^16^,^17^,^24^. More recent development of Nanoluciferase (Nluc) from a species of deep-sea shrimp has provided a significantly “brighter” BRET donor that has been harnessed for the development of “nanoBRET” assays^19^. Until now this methodology has been utilized only with chemically conjugated organic BRET acceptors, though it is theoretically amenable to use with genetically encoded protein acceptors as well^21^,^22^.

In this study, we describe a new variant of “nanoBRET” using Nluc and the red fluorescent protein mCherry. This specific BRET pair was chosen due to the large spectral separation between Nluc and mCherry emission maxima, which generates an exceedingly low background BRET ratio using the filter sets described above. This characteristic is important for generating reproducible BRET data across a large range of acceptor concentrations, particularly at concentrations far below saturation. Our empirical values for this BRET pair fit remarkably well with theoretical predictions made by the Förster equation, allowing us to calculate parameters for homotypic interactions between GLUT1 molecules with high confidence.

While we cannot determine the precise stoichiometry of higher order GLUT1 multimers using BRET alone, our data are consistent with the organizational model proposed by Carruthers *et al*. which suggests that GLUT1 monomers initially associate as N-to-N terminal dimers, and then form tetramers stabilized by homotypic interactions between juxtaposed C-termini of the four monomers (Fig. 6e)^11^,^14^. We observed the highest interaction affinity and highest BRET ratio when the donor (Nluc) and acceptor (mCherry) proteins were fused to N-termini of separate GLUT1 molecules, suggesting that N-to-N interactions occur first and are physically the closest in final complexes. The presence of tetramers allows for the possibility of BRET between GLUT1 molecules alternatively tagged on the N- and C-termini, though as expected, these interactions have lower affinity (due to later formation of tetramers) and decreased maximum BRET ratio (due to the increased distance between donor and acceptor).

Our quantitative determination of EC_50_ values for protein expression and molecular density as a function of GLUT1 multimerization provides important insights into the relevance of this phenomenon in cell types other than erythrocytes. It has been proposed that higher order complexes in erythrocytes—especially tetramers—transport glucose more efficiently than monomeric GLUT1, though the density of GLUT1 expression required for this to occur is not clear. Because GLUT1 expression on erythrocyte membranes is unusually high, accounting for an estimated 10–20% of the membrane proteome, it is possible that the mechanism of multimerization is only relevant at these remarkable surface densities^31^. Our BRET data suggest that GLUT1 oligomers begin to form at significantly lower densities of GLUT1, with EC_50_ values of about 3-fold relative to endogenous GLUT1 in 293FT cells. This corresponds to an estimated 8.4 × 10^5^ molecules of GLUT1 per cell, which is consistent with cellular expression levels reported by competitive ELISA for GLUT1 transgenic CHO cells that form higher order tetramers^32^. Given an average spherical radius of 14.5 μm for the 293FT cell line we used in this study, we estimate that a surface density of 1.27 × 10^3^ molecules/μm^2^ will result in ~50% of GLUT1 monomers being found in higher molecular weight complexes. Notably, the density of GLUT1 molecules on the surface of erythrocytes has been estimated to be about 3.1 × 10^5^ molecules of GLUT1 per cell, all of which are found in tetrameric complexes^33^. Since the surface area of human erythrocytes is about 140 μm^2^, yielding a surface GLUT1 density of 2.21 × 10^3^ molecules/μm^2^, it is unsurprising that nearly all surface GLUT1 molecules in these cells are found in higher order complexes^34^.

With our improved understanding of GLUT1 surface densities required to achieve multimerization, it seems increasingly likely that differential uptake of glucose by non-erythroid cell types may be achieved by modulating the abundance of GLUT1 on the cell surface. As surface densities approach the threshold at which higher order complexes of transporter begin to form spontaneously, glucose uptake would be expected to increase in a non-linear fashion, achieving more efficient transport into cells. This mechanism has particular relevance for cancer types that are known to upregulate GLUT1 expression in response to hypoxia—via hypoxia-inducible factor alpha, HIF1α—or signaling by other prominent oncogenic pathways, which facilitate increased glycolytic metabolism during the process of cellular transformation^35^,^36^.

It is also possible that acute regulation of GLUT1 multimerization at levels below the threshold of spontaneous aggregation explain differential glucose uptake by normal cells under starvation conditions or in response to a variety of agonists that have been previously described by our research group^37^,^38^. Of particular note is the apparent dependence of tetramers on stable disulfide bonding, which was revealed by the DTT-sensitive nature of these structures in erythrocytes^33^. Studies in mouse L929 cells, which have a low basal expression of GLUT1, have provided hints that dynamic aggregation of GLUT1—independent of increased expression—may indeed be a mechanism for activation. Nitroxyl, a compound known to induce disulfide bond formation in hydrophobic environments, activates glucose uptake 4–6 fold in these cells, as do a number of other thiol-reactive compounds such as phenylarsine oxide and cinnamaldehyde^39^,^40^,^41^. Interestingly, these same compounds fail to further activate uptake in HCLE cells, which express very high levels of GLUT1 and display high basal glucose uptake^37^. These findings imply that GLUT1 multimerization as a mechanism for regulating glucose uptake rates is relevant across a number of cells types, responding to both the abundance and redox status of the cellular environment.

## Methods

### Plasmid vector generation

Vectors containing the open reading frames for Nanoluciferase (NanoLuc, pNL1.1) and mCherry (pmCherry) were obtained commercially from *Promega* and *Clontech*, respectively. These sequences were PCR amplified using restriction site linked primers, and cloned in frame into the GATEWAY compatible pcDNA3.1-nV5-DEST or pcDNA6.2-cV5-DEST destination vectors (*Invitrogen/Life Technologies*) in place of the V5 epitope tag. This allowed us to use the GATEWAY cloning system for subsequent recombination of the mouse *Glut1* open reading frame (*Open Biosystems*, source ID 6808315) from the pCR8-TOPO entry vector into each of these vectors to create individual GLUT1 fusion proteins with Nluc or mCherry at either terminus. For the dual fusion vector (pcDNA6.2-mCherry-Glut1-Nluc), the open reading frame for mCherry was inserted in frame upstream of Glut1-Nluc in the pcDNA6.2-Glut11-Nluc vector using standard restriction cloning methods.

The fusion vectors in which mCherry and Nluc were linked together by a defined series of hexapeptide (GGSGGS) repeats were created by removing GLUT1 from pcDNA6.2-mCherry-Glut1-Nluc via restriction digest, and replacing it with a *gBlock* double-stranded DNA sequence (*Integrated DNA Technologies*) by Gibson cloning (*New England Biolabs*). This new insert contained one copy of the hexapeptide coding sequence (GGCGGCAGCGGCGGATCC) connecting the open reading frames of mCherry and NanoLuc, and was located upstream of BamH1 and Xba1 sites immediately proximal to the 5′ end of NanoLuc. Subsequent hexapeptide repeats were inserted by digesting the vectors with BamH1 and Xba1 and inserting in an annealed, double-stranded oligonucleotide encoding a single hexapeptide repeat flanked by overhangs homologous to the BamH1 and Xba1 restriction sites. Insertion of this oligonucleotide extended the repeat by a single unit in frame with the mCherry and Nluc proteins and re-introduced new 3′ BamH1 and Xba1 sites, while at the same time destroying the 5′ BamH1 site. Repeated iterations of this protocol allowed for creation of a series of linker vectors in which mCherry and Nluc were connected by a linker of 1–10 repeats of the hexapeptide.

The stable transposable element vectors based on the Sleeping Beauty system were created by subcloning the expression cassettes for mCherry-GLUT1 and Nluc-GLUT1 into the pT2-HB vector (obtained from Dr. Perry Hackett via *Addgene*, #26557)^29^. The original cytomegalovirus promoters from the pcDNA3.1 expression vectors (see above) were subsequently excised and replaced with either the constitutive human elongation factor 1-alpha promoter (EF1α, for Nluc-GLUT1) or the Tet3G-responsive element promoter (TRE3G, for mCherry-GLUT1). We also subcloned the open reading frame for Tet3G cassette (from the pCMV-TET3G, *Clontech*) into pT2-EF1α to create a constitutively expressing stable vector for tetracycline/doxycycline inducible expression of mCherry-GLUT1. Partial maps for these three vectors are shown in Fig. 6a. Stable insertion of each cassette into cells was carried out by co-transfection of the pT2 series vectors with pCMV(CAT)T7-SB100 (obtained from Dr. Zsuzsanna Izsvak via *Addgene*, #34879), which encodes the SB100X transposase^30^.

### Cell culture and transfections

The 293FT-HEK cells (*Invitrogen*) used in this study were cultured in Dulbecco’s Modified Eagle Medium (DMEM, *Gibco*), which was supplemented with 10% fetal bovine serum (*Atlanta Biologicals*). Cells were split three times per week to maintain log-phase growth, and seeded at specific densities after counting with a standard hemocytometer. Transient transfections into 293FT-HEK cells were performed using LipoD293 cationic lipid reagent (*SignaGen Laboratories*) at a ratio of 3 μL of reagent per 1 μg of plasmid DNA. Transfections into cells plated to 96-well plates (see below) were performed in a final volume of 100 μL per well, whereas transfections into cells in 6-well plates were done in a final volume of 2 mL. In both cases, media was subsequently changed 18–24 hours after transfection to remove excess cationic lipid from the media. Stable insertion of transposable elements was selected for by culture of cells in media containing 2.0 μg/mL of puromycin (*Sigma-Aldrich*).

### BRET assay measurements

BRET assays were uniformly performed in white-walled, microclear-bottom 96-well plates into which 293FT cells were seeded at a density of 1.0 × 10^4^ per well (*Greiner Biosciences*). Cells were transfected in triplicate with expression plasmids (50 ng per well) at 18–24 hours post-seeding. The following day (~24 hours post-transfection), media was removed and replaced with fresh complete media. At 48 hours post-transfection media was again removed and replaced with serum and phenol red-free DMEM media, and cells were allowed to equilibrate for one hour under normal culture conditions. The cells were subsequently treated with 5 nm furimazine (*Promega*) and immediately measured for emission of light at 410 and 610 nanometers using a Synergy Hybrid H1 plate reader (*BioTek*) equipped with a 410/80 and 610-long pass luminescence filter set. The corrected BRET ratio was determined by subtracting the basal 610/410 filtered luminescence value of GLUT1-Nluc alone (*I*_*D*_) from the 610/410 ratio of cells also expressing mCherry-fusion acceptors (*I*_*DA*_) at various levels.

For BRET assays performed in parallel with flow cytometry to allow for calculation of mCherry expression density, 1.0 × 10^6^ cells were transfected in 6-well plates with volumes scaled according to the surface area ratio of 96-well to 6-well plates (1:100). At 24 hours after transfection, the cells were reseeded in parallel to a new 6-well plate and in triplicate to a 96-well assay plate at densities of 1.0 × 10^6^ and 2.0 × 10^4^ cells per well, respectively. The BRET assays were performed 18–24 hours after replating as indicated above.

### 2-Deoxy-glucose uptake assays

HEK-293FT cells were plated to 6-well dishes at a density of 5.0 × 10^4^ cells/well and transfected 24 hours later with 1.0 μg/well of plasmid DNA for each GLUT1 fusion protein. After 48 hours, the cells were detached and replated to 24-well dishes at a density of 5.0 × 10^4^ cells/well and allowed to adhere and equilibrate to culture conditions for 18–24 hours. Glucose uptake was measured using the radiolabeled glucose analog 2-deoxyglucose (2DG), which was added with ^14^C-mannitol as a control for cell membrane integrity. Briefly, the media was replaced with 0.3 mL of glucose-free HEPES buffer [pH 7.4] (140 mM NaCl, 5 mM KCl, 20 mM HEPES, 2.5 mM MgSO_4_, 1 mM CaCl_2_, 2 mM sodium pyruvate, 1 mM mannitol) supplemented with 1.0 mM (0.3 μCi/mL) 2-DG (1, 2-^3^H) and 1.0 mM (0.02 μCi/mL) mannitol (1-^14^C). After a 10 minute incubation, cells were washed twice with cold glucose-free HEPES. The cells were digested in 0.25 mL 0.3 M NaOH prior to measuring the ^3^H-2DG uptake and ^14^C-mannitol background signal. Quadruplicate ^3^H-2DG uptake values corrected for ^14^C-mannitol binding were averaged and tested for significance using student’s T-test.

### Flow cytometry

Transfected 293FT cells were replated to 6-well dishes and allowed to adhere and equilibrate to culture conditions for 18–24 hours. Cells were rinsed with PBS and detached from the plate in 1 mL of cold versene (*Invitrogen/Life Technologies*). Cells were subsequently filtered to achieve a single cell suspension, and immediately assayed on a FACScalibur flow cytometer (*Becton Dickinson*). Each cell population was gated according to forward and side scatter profile to identify intact cells, which were then measured on the FL2 channel to quantify mCherry expression. No fewer than 2.0 × 10^4^ cells were captured for each sample, and each condition was measured in triplicate to obtain a mean fluorescent intensity (MFI) for the population. Mean fluorescent intensities were translated into molecular equivalents of soluble fluorophore (MESF) using calibrated mCherry beads (*Clontech*) with defined MESF values. The beads were run for each assay replicate using the same laser settings that were used to quantify mCherry expression in transfected cells.

### Immunoblotting

Transfected cells in 6-well format were rinsed once with PBS and directly scraped into 0.1 mL lysis buffer (20 mM Tris [pH 7.5], 150 mM NaCl, 1 mM EDTA, 1 mM EGTA, 2.5 mM sodium pyrophosphate, 1 mM sodium glycerophosphate, 1 mM sodium orthovanadate, 0.5% NP40, 0.1% Brij35, 0.1% sodium deoxycholate) supplemented with fresh protease inhibitor cocktail (Sigma-Aldrich). The lysate was homogenized by sonicating for 5 seconds while incubating on ice, and then cleared by centrifugation at 10,000 × g for 5 minutes in a chilled centrifuge at 4 °C. Soluble protein was removed to a fresh tube on ice, quantified by Bradford assay and denatured for 10 minutes at 95 °C in a dry bath. The samples were then deglycosylated with recombinant PNGaseF (prepared from *E.coli* using the pOPH6 vector from Dr. Shaun Lott, obtained via *Addgene*, #40315)^42^, diluted with 5× Laemmli sample loading buffer, and then separated by SDS-PAGE on an 8% Tris-glycine gel. The gel was transferred overnight at 50 mA and 4 °C to a nitrocellulose membrane in Tris-glycine transfer buffer. The membrane was blocked with 3% non-fat dry milk in Tris-buffered saline with 0.05% Tween 20 (TBST), and then incubated overnight with primary antibodies diluted 1:1000 in 3% BSA/TBST at 4 °C. Primary antibodies were washed off in three consecutive rinses in TBST for 5 minutes at room temperature, and the membrane was subsequently incubated in TBST containing at 1:25,000 dilution of secondary antibodies (goat-anti-mouse-DyLight 700 and goat-anti-rabbit-DyLight 800, Cell Signaling Technologies). After a final three rinses for 5 minutes each in TBST, the membranes were scanned and quantified on a Li-Cor Odyssey infrared scanner. The polyclonal rabbit antibody targeted to the C-terminus of GLUT1 was purchased from Epitomics/Abcam, while the mouse monoclonal antibody to β-actin was purchased from Sigma-Aldrich.

### Graphing and statistical analysis

Data for each experiment were exported into the Prism software package (Mac version 6, *GraphPad Software*) for graphing and statistical analysis. Data were best fit to standard hyberbolic or sigmoidal curves with correlation coefficients indicated in each legend. EC_50_ values with 95% confidence intervals were obtained from triplicate or quadruplicate experiments as indicated and, when relevant, analyzed for significance using student’s T-test.

## Additional Information

**How to cite this article**: Looyenga, B. *et al*. Determination of GLUT1 Oligomerization Parameters using Bioluminescent Förster Resonance Energy Transfer. *Sci. Rep.* **6**, 29130; doi: 10.1038/srep29130 (2016).

## Supplementary Material

Supplementary Information

## Figures and Tables

**Table 1:** Theoretical BRET Values for NanoLuciferase Donor and Fluorescent Protein Acceptor Pairs.

Fluorescent Protein	Extinction Coefficient (M^−1^cm^−1^) ε	Fluorescence Quantum Yield Φ	Brightness (ε•Φ)	Spectral Overlap (x10^14^)	Theoretical Förster Radius (Å)	Acceptor Emission Maximum (nm)	Peak Emission Shift (nm)*	Background Nanoluc Signal**
mCherry	72000	0.22	15840	4.19	37.1	610	160	0.06
mPlum	41000	0.01	410	2.4	33.8	649	199	0.06
mStrawberry	90000	0.29	26100	6.64	40.1	596	146	0.11
mTangerine	38000	0.3	11400	2.9	34.9	585	135	0.14
mOrange2	58000	0.6	34800	5.39	38.7	565	115	0.34
mKO	51600	0.6	30960	4.68	37.8	559	109	0.39
mBanana	6000	0.7	4200	0.73	27.7	553	103	0.43
Citrine (YFP)	77000	0.76	58520	7.66	41.0	529	79	1.21
Emerald GFP	57500	0.68	39100	13.7	45.2	509	59	1.70
mHoneydew	17000	0.12	2040	5.47	38.8	562	112	2.11
Cerulean (CFP)	43000	0.62	26660	9.86	42.8	475	25	4.54

(*the peak emission shift was calculated by subtracting the Nluc emission maximum [450 nm] from the acceptor protein emission maximum; **the theoretical background BRET ratio was calculated by taking the ratio of light emitted by Nluc alone at the acceptor emission maxima divided by the measured donor wavelength [410 nm]).
