# Supplementary material for: Determination of GLUT1 Oligomerization Parameters using Bioluminescent Förster Resonance Energy Transfer
Source: Sci Rep. 2016 Jun 30;6:29130. doi: 10.1038/srep29130 (PMC4928127; doi:10.1038/srep29130)
Supplement: Supplementary Information [file srep29130-s1.pdf]

Supplemental Information

Determination of GLUT1 Oligomerization Parameters using Bioluminescent Förster Resonance Energy Transfer

Brendan Looyenga[1*], Calvin VanOpstall[1], Zion Lee[1], Jed Bell[1], Evans Lodge[1], Katherine Wrobel[1], Eric Arnoys[1] and Larry Louters[1]

[1] Calvin College, Department of Chemistry & Biochemistry, 3201 Burton St SE, Grand Rapids, MI, 49546

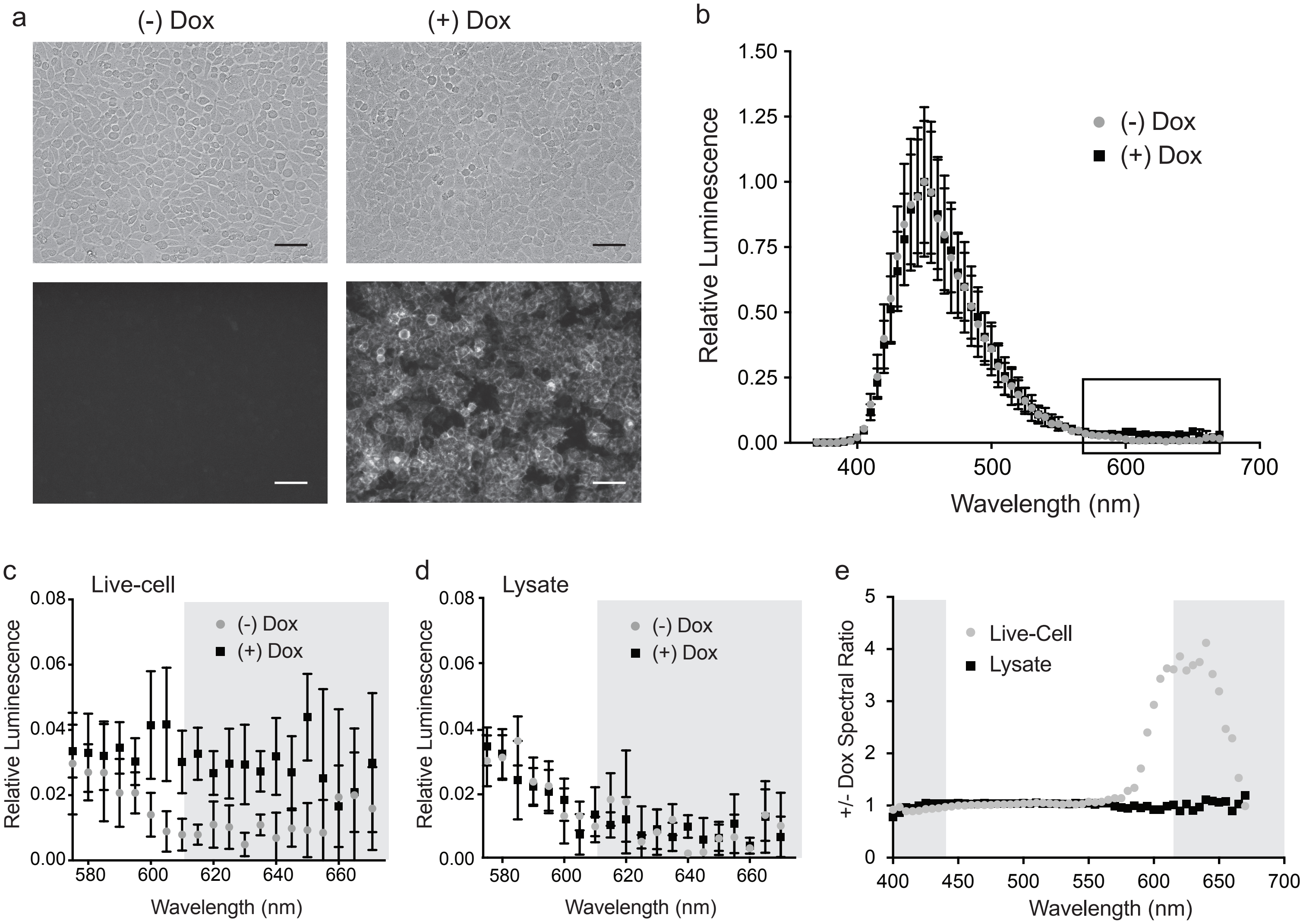

Figure S1 – Spectral analysis of nanoBRET with Nanoluciferase and mCherry Glut1 fusion proteins. **(a)** Cells that constitutively express Nluc-GLUT1 from the human EF1α promoter and inducibly express mCherry-GLUT1 with the TET3G system were cultured without doxycycline or with 500 ng/mL of doxycycline for 48 hours. The two cell populations were imaged under brightfield illumination and red LED (586/15 nm) excitation using a FLoid Cell Imaging Station to demonstrate inducible mCherry-GLUT1 expression. Scale bars = 100 microns. **(b)** The live-cell luminescent emission spectrum of cells +/- doxycline stimulation was captured at 5 nm intervals from 370-670 nm using the monochromator on a BioTek H1 synergy plate reader. The raw spectral data from each sample was normalized to peak Nluc emission (450 nm) to create a relative luminescent spectrum. Error bars represent standard deviations of four biological replicates for each condition. **(c)** Live-cell spectral emission data in the area of the curve corresponding to mCherry (box in panel b). Resonant energy transfer from Nluc to mCherry is demonstrated by the increased signal intensity for wavelengths above 580 nm when mCherry-GLUT1 is expressed (+Dox). The gray background highlight indicates the region of emission captured by the 610-long pass filter used for filtered luminescence measurement of the mCherry signal in standard BRET assays. **(d)** Spectral measurements were repeated after cells were lysed with a buffer containing membrane-disrupting detergents (see “Immunoblot” methods for composition). Increased signal intensity for wavelengths above 580 nm is lost in cells expressing mCherry-Glut1 (+Dox), indicating disruption of resonant energy transfer from Nluc-Glut1 to mCherry-Glut1 by dissociation of membrane structure. **(e)** The ratio of luminescent signals +/- doxycycline was calculated at 5 nm intervals for live- and detergent-lysed cells. Intact cells display a clear increase in signal in the mCherry emission spectra, while this signal is lost when the membrane is disrupted. The gray background highlights indicate regions of emission captured by the 410/80 filter (for Nluc) and 610-long pass filter (for mCherry) used for filtered luminescence BRET assays.
